# Does authentic leadership promote higher job satisfaction in public versus private organizations? Exploring the role of vigor and engagement

**DOI:** 10.1016/j.heliyon.2023.e12906

**Published:** 2023-01-10

**Authors:** Daniel Cortés-Denia, Octavio Luque-Reca, Esther Lopez-Zafra, Manuel Pulido-Martos

**Affiliations:** a Department of Psychology. Universidad de Jaén, Jaén, Spain; b Department of Psychology, Universidad Rey Juan Carlos, Madrid, Spain

**Keywords:** Authentic leadership, Job satisfaction, Private/public organizations, Vigor at work, Work engagement

## Abstract

**Background:**

Several studies have suggested that leaders showing high levels of authentic leadership increase workers' overall job satisfaction, which is composed of different aspects with some distinctions among them. Furthermore, the implication of affective and motivational variables, such as vigor and engagement at work, respectively, have not been jointly considered for analysing the differences and similarities between public and private organizations. Thus, this study aims to analyze whether vigor at work and engagement at work play a mediating role between authentic leadership and different aspects of job satisfaction, considering both public and private organizations, to propose a *trans*-organizational model.

**Method:**

In this cross-sectional and cross-sectoral study, 1029 workers in private (*n* = 619) and public (*n* = 410) organizations from Spain participated completing a questionnaire.

**Results:**

Structural equation modelling was used to perform a multigroup mediation analysis (public versus private organizations) in which the invariance between groups was previously explored. The model showed a good fit to the data in which authentic leadership affected the dimensions of job satisfaction both directly and indirectly (through vigor at work and engagement). However, authentic leadership had a greater positive effect on vigor at work for private organizations; whereas work engagement was not significantly related to the job satisfaction dimension related to legal aspects for public organizations.

**Conclusion:**

The vigor at work and work engagement were important variables to explain the authentic leadership-job satisfaction relationship in both private and public organizations. Nevertheless, the relationship between work engagement and the dimensions of job satisfaction was different for both organizations.

## 1 Introduction

Although the relationship between a leader's leadership style and employees’ job satisfaction has been previously studied in Positive Organizational Psychology, it is important to delve into both the knowledge of the variables capable of mediating these relationships and the analysis of which components of job satisfaction are further promoted. In addition, within this topic, attention must be paid to the possible differences between different work contexts. Thus, by comparing public versus private sector workers, this research seeks to explore the possible mediating role that a motivational variable and a personal resource, such as work engagement and vigor at work, respectively, play in the relationship between leadership and specific facets of job satisfaction.

### 1.1 Authentic leadership

Leadership is a key factor in organizations [1,2]. It allows incentivizing and motivating workers through the mobilization of various resources, further improving their performance and thus achieving organizational objectives [3]. Therefore, leaders exert an influence on the work environment and affect the generation of demands and resources and, ultimately, the production of different work results, such as well-being and performance [4]. In modern organizations, there is a need for positive leadership that focuses on mutual recognition. People require leaders who symbolize principles and straightforwardness and who embody ethical principles [5]. One positive leadership style is *authentic leadership* (AL), which is defined as the “process that draws from both positive psychological capacities and a highly developed organizational context, which results in both greater self-awareness and self-regulated positive behaviors on the part of leaders and associates, fostering self-development” (p. 243) [6]. This leadership style may result in a social job resource [7] that is relevant to addressing the different demands of and challenges to organizations. In doing this, leaders create a perfect fit between the values, behaviors, and actions that constitute this approach [6] promoting positive emotions and actions in employees as better performance or well-being [8].

### 1.2 The authentic leadership-job satisfaction relationship in public and private organizations

Several studies have suggested that high levels of AL are sufficient to increase workers' job satisfaction [[9], [10], [11], [12]]. Job satisfaction has often been considered a global construct [13], which is composed of relatively homogeneous but sufficiently different aspects. Therefore, job satisfaction is a multidimensional construct that includes a multitude of psychological states and beliefs [14], and may be divided into dimensions or facets [15]. Furthermore, there may be subtleties in how different characteristics in the workplace may influence one or more dimensions of job satisfaction [16], which may lead to results that are unclear or inconclusive if analyzed globally. For instance, workers may feel satisfied with components such as the content of their job, their work environment, or their responsibilities; but at the same time, they could be unsatisfied concerning to mobility within the organization or their potential for growth [17,18].

One of the environments in which differences in employee satisfaction with their leaders have been proven to exist is related to the organizational context: public vs. private [19,20]. The real challenge is to compare both contexts regarding differences and similarities [21]. Various studies have analyzed whether there are differences in the levels of job satisfaction between the two contexts. However, there is no agreement in terms of the results. Wang et al. [22] found differences in job satisfaction between public and private employees. Specifically, workers in the public sector were less satisfied than workers in the private sector. However, other studies found the public sector to have the most satisfied workers [23,24]. Due to their differences, employees in public vs. private organizations place distinct values on different motivational factors [25,26], which affects their satisfaction. Therefore, leaders face the challenge of motivating and enhancing employee performance concerning to the organizational context and employee needs [27]. This organizational context could influence different resources in a variety of ways and hence explain discrepancies in job satisfaction results. However, studies addressing AL and job outcomes have mainly focused on one specific context (either private or public), whereas comparing the two domains could help understand the differing results.

### 1.3 Mediators in the authentic leadership-job satisfaction relationship

From job demands-resources theory (JD-R; [28]), expanding the original model [29,30], the role of personal and labor resources in understanding and explaining workers’ well-being is included and emphasized. Specifically, AL, as a job resource, could produce an increase in the experienced levels of well-being and work engagement of the workers [31,32]. In this sense, authentic leaders’ behaviors mitigate job dissatisfaction and reduce the risk of burnout [33] building a positive work environment that makes it more likely to engage and perform better [34]. In fact, the research has also focused on the implications of AL on work engagement and job satisfaction [35].

Work engagement was defined by Schaufeli et al. [36] as “a positive, fulfilling, work-related state of mind that is characterized by vigor, dedication, and absorption” (p. 74). Work engagement is composed of vigor, which refers to the level of energy, effort, and resilience displayed in the workplace; dedication, which focuses on feelings of enthusiasm, pride, and challenge; and absorption, which represents the state of concentration when performing a task. Several studies have emphasized the importance of engagement [37,38] or have even analyzed the influence of both variables (AL and engagement) on job satisfaction [39,40], but without considering personal resources, who could have an important role in that relationship. Actually, following JD-R theory, there is a reciprocal relationship between both labor and personal resources in explaining the levels of well-being of workers, specifically on work engagement [41].

Xanthopoulou et al. [42] suggested that labor resources encourage the development of personal resources and that the latter partly mediates the relationship between labor resources and engagement. In this vein, given that AL has influenced employees' energy sources [43], a resource related to energy could be maintained or benefitted through different motivational variables. Therefore, we could consider vigor at work, an affective personal resource, to be a mediator in the relationship between AL and work engagement as a motivational variable. Specifically, vigor at work has been defined as a positive affective state characterized by experiencing feelings of physical strength, which represent the physical capacities of the individual; emotional energy, such as the ability to show and express empathy and compassion to other people; and cognitive liveliness, which is described as the flow of thought processes and mental agility [44,45].

To date, there is only a small amount of research addressing the combination of vigor at work with work engagement to demonstrate the lack of overlap between the variables or even to acknowledge their various contributions to different work results [46,47]. In fact, this construct differs from work engagement, empirically showing discriminant validity regarding engagement [46]. Also, Cortés-Denia et al. [48]; in their systematic review, concluded that vigor at work and work engagement are complementary constructs. In particular, vigor at work, which manifests as a positive affect, constitutes a personal resource, whereas work engagement, which is a motivational outcome, is affected by both personal and job resources. Additionally, in line with Borst et al. [49], studies, which explore the role played by factors such as work engagement and vigor at work in the public sector are limited. Moreover, authors such as Bakker and Albrecht [50] pointed out the need for future studies to analyze these variables by differentiating between the public and private sectors.

Thus, this research contributes to knowledge by addressing some novel aspects: i) to analyze the relationship between AL and the different facets of job satisfaction, instead of studying the latter globally; ii) to analyze for the first time the possible mediating role that an affective variable (i.e., vigor at work) and a motivational variable (i.e., work engagement) exert jointly in the AL-job satisfaction relationship; and iii) to explore whether these relationships of dependency and mediation differ in workers from the public sector compared to the private sector, due to the discrepancies found in engagement and job satisfaction in public/private organizations.

### 1.4 Objective and hypothesis

Using structural equation model (SEM) methodology, this study aims to analyze whether vigor at work and engagement at work play a mediating role between AL and different aspects of job satisfaction. Furthermore, we consider both public and private organizations to propose a *trans*-organizational model. Specifically, we propose the following.

Hypothesis 1 Vigor at work mediates the relationship between AL and work engagement.

Hypothesis 2 Vigor and engagement at work mediate the relationship between AL and job satisfaction.

Hypothesis 3 Job satisfaction differs between private and public organizations as a result of these mediation effects. Specifically, vigor and engagement at work could have different impacts according to context.

## 2 Method

### 2.1 Participants

Participants were 1029 employees working in private and public organizations in different provinces of Spain. Table 1 presents the demographic characteristics of the participants, both globally and by the public or private sector.

**Table 1:** Demographic characteristics of the study participants.

	Private organizations (*n* = 619)		Public organizations (*n* = 410)		Total (*n* = 1029)	
Gender	*n*	%	*n*	%	*n*	%
Male	316	51.1	141	34.4	457	44.4
Female	303	48.9	269	65.6	572	55.6
	Mean	SD	Mean	SD	Mean	SD
Age (years)	38.2	11.7	43.6	11.9	40.3	12.1
Tenure in the organization (years)	8.8	10.1	16.0	13.1	11.7	11.9

Note: SD = standard deviation.

### 2.2 Missing data

Of the 1087 employees initially recruited, 58 (0.45%) had missing values in at least one of the variables. The result of Little's test (χ^2^ = 2185.86, df = 1983, *p* < 0.01) suggested that these data were not *missing completely at random* (MCAR). The rate of missing data was substantially less than 5% of cases, which does not imply significant problems, with a lack of statistical power or biases in the parameter estimates [51]. Thus, a *listwise deletion* approach was taken, resulting in the final sample. This procedure is commonly used with SEMs and is appropriate in cases involving large sample sizes and a low number of missing values [52].

### 2.3 Measures

The following scales were included (see Table 2 for examples of each dimension, measurements of validity, and reliability values).

**Table 2:** Descriptive statistics, reliability and validity coefficients by private vs. public organizations.

	Private organizations		Public organizations		Coefficients	
Variable	Mean	SD	Mean	SD	CR	AVE
*Relational transparency (AL) (0 = never; 4 = always)*					0.86	0.56
Exactly say what means to	2.87	1.07	2.80	1.08		
Admit mistakes	2.45	1.27	2.52	1.23		
Encourage to speak	2.60	1.26	2.68	1.17		
Let others know who truly is	2.92	1.11	2.80	1.18		
Openly share feelings	2.56	1.16	2.51	1.12		
*Internalized moral perspective (AL) (0 = never; 4 = always)*					0.85	0.58
Beliefs are consistent with others	2.60	1.11	2.59	1.08		
Make decisions based on core values	2.84	1.07	2.74	1.08		
Ask to assume core values	2.55	1.15	2.42	1.22		
Make decisions based on high ethical conduct	2.45	1.21	2.51	1.18		
*Balanced processing (AL) (0 = never; 4 = always)*					0.88	0.70
Solicit views that challenge positions	2.15	1.27	2.16	1.16		
Analyze data before making a decision	2.58	1.16	2.63	1.09		
Listen to different points of view	2.47	1.24	2.51	1.13		
*Self-awareness (AL) (0 = never; 4 = always)*					0.91	0.72
Seek feedback to improve interactions	2.42	1.28	2.43	1.15		
Describe how others view capabilities	2.40	1.18	2.40	1.07		
Know when to reevaluate position	2.36	1.15	2.36	1.08		
Understand actions impact others	2.43	1.18	2.39	1.10		
*Physical strength (VW) (1 = never or hardly ever; 7 = always or everyday)*					0.94	0.76
Full of pep	4.91	1.28	4.85	1.32		
Physical energy	4.86	1.27	4.74	1.31		
Strong	4.97	1.24	4.93	1.26		
Active	5.43	1.17	5.28	1.19		
Vitality	5.35	1.19	5.32	1.21		
*Cognitive liveliness (VW) (1 = never or hardly ever; 7 = always or everyday)*					0.89	0.72
Think rapidly	5.48	1.16	5.44	1.07		
Contribute new ideas	5.41	1.23	5.33	1.14		
Be creative	5.36	1.28	5.22	1.23		
*Emotional energy (VW) (1 = never or hardly ever; 7 = always or everyday)*					0.88	0.65
Show warmth to others	5.70	1.25	5.83	1.15		
Be sensitive to others’ needs	5.84	1.06	5.84	0.94		
Invest emotionally in others	5.70	1.18	5.71	1.13		
Be sympathetic to others	5.92	1.12	6.00	1.01		
*Engagement (0 = never or hardly ever; 6 = always, everyday)*					0.92	0.55
Feel like going to work in mornings	3.61	1.79	3.95	1.73		
Feel strong and vigorous	4.67	1.20	4.65	1.20		
Feel bursting with energy	4.63	1.20	4.67	1.26		
Proud of the work I do	4.65	1.42	5.04	1.20		
Job inspires me	3.72	1.70	4.24	1.54		
Enthusiastic about my job	4.21	1.50	4.63	1.38		
Time flies	3.51	1.62	3.66	1.62		
Immersed in my work	4.26	1.50	4.68	1.26		
Happy when I am working	3.86	1.57	4.32	1.51		
*Satisfaction with environment (1 = very dissatisfied; 7 = very satisfied)*					0.76	0.53
Cleanliness, hygiene, and health	5.56	1.38	5.45	1.46		
Physical environment	5.34	1.38	5.15	1.49		
Temperature	4.79	1.69	4.78	1.70		
*Satisfaction with supervisors (1 = very dissatisfied; 7 = very satisfied)*					0.94	0.71
Personal relations with superiors	5.43	1.45	5.39	1.44		
Supervision	5.15	1.51	5.12	1.47		
Proximity and monitoring frequency	5.04	1.51	4.96	1.43		
Way to judge your tasks	5.04	1.71	4.97	1.61		
Equality and fair treatment	5.05	1.71	4.85	1.76		
Support from superiors	5.07	1.65	4.97	1.73		
*Satisfaction with the legal aspects (1 = very dissatisfied; 7 = very satisfied)*					0.88	0.79
Legal and regulatory compliance	5.14	1.76	5.06	1.79		
Labor negotiations	4.82	1.76	4.51	1.82		

Note: AL = authentic leadership; VW = vigor at work; SD = standard deviation; CR = composite reliability; AVE = average variance extracted.

*Authentic leadership*. This variable was assessed using the Authentic Leadership Questionnaire (ALQ; Walumbwa et al. [53]; Spanish version by Moriano et al. [54]). This 16-item questionnaire with a 5-point Likert response format contains the following four subscales: relational transparency (5 items), internalized moral perspective (4 items), balanced processing of information (3 items), and self-awareness (4 items). Good internal consistency for the original subscales was reported [53] ranging from 0.76 to 0.92.

*Vigor at work.* The Shirom-Melamed Vigor Measure (SMVM; Shirom [44]; Spanish version by Pulido-Martos et al. [55]) was used to measure vigor at work. This 12-item scale uses a 7-point Likert response format and comprises three subscales: physical strength (5 items), cognitive liveliness (3 items), and emotional energy (4 items). The authors of the scale [56] found high internal consistency for the three subscales (ranging from 0.85 to 0.93).

*Work engagement*. The 9-item Utrecht Work Engagement Scale (UWES-9; Schaufeli et al. [57]; original Spanish version by Schaufeli et al. [36]) was used to measure engagement. This scale uses a 7-point Likert response format and comprises three dimensions: vigor (3 items), dedication (3 items), and absorption (3 items). However, researchers can also consider one dimension using the total nine-item score as an indicator of work engagement [57]. In this study, engagement is considered overall, and thus, it is appropriate to evaluate this factor with a one-dimensional model [58]. The reliability of the total scale was high, ranging between 0.85 and 0.92 in different studies conducted by the authors of the original version [57].

*Job satisfaction* was measured using the S10/12 Job Satisfaction Questionnaire [59]. The questionnaire uses a 7-point Likert response format and comprises three subscales measuring different aspects of work satisfaction. Specifically, the questionnaire measures three dimensions. Satisfaction with the environment (3 items), refers to the physical aspects of the work environment and has shown adequate internal consistency (0.72). Satisfaction with supervision (6 items), refers to the relations, support, and supervision received from superiors as well as equality and fairness of treatment and has demonstrated good internal consistency (0.89). And satisfaction with legal aspects (2 items), which refers to the means and procedures for negotiating work benefits and the degree of their compliance and has shown adequate internal consistency (0.74). Due to the multidimensionality of work satisfaction, each dimension is considered independently.

### 2.4 Procedure

The researchers contacted different organizations both public and private and applied for permission to request employee participation. Then, employees from different organizations were contacted, and those who consented to voluntarily participate were approached in their workplace by psychology undergraduate students involved in a research seminar. The students received exhaustive training in the procedure, recruitment and distribution of the survey, following recommendations to check the answers’ veracity (see Wheeler et al. [60]. Participation took place in 2021. This study followed the protocol approved by the Ethics Committee of the University of Jaén, Spain (Ref. NOV.19/1.PROY). The criteria for participation were status as a worker in a public or private organization and the requirement of working under the supervision of a direct leader for at least six months.

### 2.5 Statistical analyses

To perform two-phase analyses, SEM methodology was employed. In the first phase, the measurement model is tested to analyze the validity, reliability and invariance of the factor structure of the variables in both groups (public versus private workers), as well as to explore differences in the latent means between these groups. In the second phase, the structural model is tested using a multigroup analysis, to explore the relationships among the variables and analyze whether such relationships vary between the two groups of workers. All analyses were conducted using EQS 6.4 software for Windows.

In the first phase, as a preliminary step, a confirmatory factor analysis (CFA) was carried out, in which a model composed of the following 11 first-order factors was explored: relational transparency, internalized moral perspective, balanced processing of information, self-awareness, physical strength, cognitive liveliness, emotional energy, engagement, satisfaction with the environment, satisfaction with supervision and satisfaction with legal aspects. CFA was performed to analyze the reliability and convergent validity of the latent variables using the composite reliability (CR) and average variance extracted (AVE), respectively. CR results over 0.70 and AVE scores over 0.50 were considered adequate [52]. In addition, CFA was used to evaluate the general adjustment of this factorial model using the following global fit indices: the robust comparative fit index (CFI) and the robust root-mean-square error of approximation (RMSEA). Adjustment criteria values were greater than or equal to 0.90 for CFI and less than 0.05 for RMSEA [61,62].

Subsequently, within this first phase, mean differences between employees according to the organization (i.e., private vs. public) were assessed by multigroup latent mean analysis of the latent variables (first- and second-order factors), overcoming the limitations inherent in the use of traditional analyses (i.e., *t*-test or MANOVA) [63]. All measurement models tested in this analysis included the previous 11 first-order factors. Four of these factors (relational transparency, internalized moral perspective, balanced processing of information, and self-awareness) were grouped into a second-order factor called AL and three of the factors (physical strength, cognitive liveliness and emotional energy) were grouped into a second-order factor termed vigor at work. Thus, several hierarchical invariance tests were performed to compare means at the factor level. Specifically, we tested the following analyses across multiple groups in this order: configural invariance, metric invariance, and scalar invariance. CFA was performed to test the fit of the measurement model to the data for each of the groups (private vs. public organizations). The simultaneous adjustment of the measurement model was tested by multigroup analysis. This configural model serves as a baseline model to test by imposing restrictions by stages regarding different types of invariance [63,64]. Next, analyses were carried out to show the latent mean differences between employees of private and public organizations following [65] proposal (MACS; means and covariance structure). The means of the factors were set to 0 for the group of public organizations taken as a reference and were established as free parameters for the group of private organizations. This approach permitted the use of *z* statistics to test the level of significance of the differences and to report the effect size using the Cohen *d* index [66].

In the last phase, the possible differences between groups in the established structural model were analyzed. As a baseline model, the structural model was chosen, and a multigroup analysis was performed. In a later step, the measurement invariance across groups was evaluated by setting the restrictions on the factor loadings derived from the previous phase. Finally, group restrictions were established by setting all structural paths of the model as equal. For the evaluation of the different models, a robust type analysis was performed for the biases in the kurtosis indicators using the global fit indices mentioned above [61,62]. To make conclusions regarding the different types of invariance, when comparing nested models, it was decided not to rely exclusively on the results of the tests of differences in the values of corrected χ^2^ due to associated concerns [67,68]. Additionally, a further increase in the CFI index was considered for values below 0.01 [69]. Lagrange multiplier (LM) test modification indices were used to release the restrictions established in the comparison of groups when necessary.

## 3 Results

### 3.1 Descriptive statistics

In Table 2, the means, standard deviations, and response ranges are shown for the variables of the study organized according to groups of employees (private organizations vs. public organizations). Moreover, CR and AVE values for each dimension are displayed.

### 3.2 Phase 1. Latent mean analysis

Preliminarily, CFA showed an adequate fit of the factorial model for the total sample (Satorra–Bentler χ2 statistic [S-B*χ*^2^] = 3439.761, *df* = 1105, *p* < 0.001; CFI = 0.918; RMSEA = 0.045 [90% CI from 0.044 to 0.047]) and good CR and AVE values for the first-order factors were obtained (see Table 2).

The measurement model that best fit both groups of workers included hierarchical structures for the vigor at work (1 second-order and 3 first-order factors) and AL (1 second-order and 3 first-order factors) constructs. Moreover, the model included the following factors: engagement, satisfaction with the work environment, satisfaction with supervision, and satisfaction with legal aspects. The first two rows of Table 3 include the overall adjustment indices for the measurement model, which was tested in a multigroup model by demonstrating its configural invariance and taking it as a baseline model for subsequent invariance tests, which were conducted separately for groups of employees in private and public organizations. See Table 3 for the results of the adjustment for both the configural model and the other nested models.

**Table 3:** Fit indices of the measurement model for invariance tests related to multigroup analyses.

	S-Bχ^2^	*df*	RMSEA (90% CI of RMSEA)	CFI	ΔS-Bχ^2^^a^	ΔCFI
Private organizations (*n* = 619)	2444.73	1058	0.046 (0.044–0.048)	0.918		
Public organizations (*n* = 410)	2004.39	1058	0.047 (0.044–0.050)	0.919		
Configural model	4464.85	2116	0.047 (0.045–0.048)	0.918		
Measurement invariance (first-order factor loadings invariant)	4516.44	2152	0.046 (0.044–0.048)	0.918	48.75 (Δ*df* = 36)	0.000
Measurement invariance (first- and second-order factor loadings invariant)	4532.96	2159	0.046 (0.044–0.048)	0.918	65.05∗ (Δ*df* = 43)	0.000
Partial measurement invariance (first- and second-order factor loadings invariant)	4515.68	2154	0.046 (0.044–0.048)	0.918	47.52 (Δ*df* = 38)	0.000
Scalar invariance (first- and second-order factor loadings; observed variable intercepts invariant)	4682.96	2196	0.047 (0.045–0.049)	0.918	244.93*** (Δ*df* = 80)	0.000
Partial scalar invariance (first- and second-order factor loadings; observed variable intercepts invariant)	4548.34	2178	0.046 (0.045–0.048)	0.918	68.31 (Δ*df* = 62)	0.000
Scalar invariance (first- and second-order factor loadings; observed variable and latent factor intercepts invariant)	4681.54	2187	0.048 (0.046–0.049)	0.914	268.75*** (Δ*df* = 71)	0.004
Partial scalar invariance (first- and second-order factor loadings; observed variable and latent factor intercepts invariant)	4672.04	2185	0.048 (0.046–0.049)	0.914	255.85*** (Δ*df* = 69)	0.004

Note: CFI = comparative fit index; CI = confidence interval; *df* = degrees of freedom; RMSEA = root mean-square error of approximation; S-Bχ^2^ = Satorra–Bentler χ^2^ statistic.

*p < 0.05; *** *p* < 0.001.

a Corrected value.

In the first step (results in Table 3; rows 4–6), metric invariance was tested, first by setting the factor loadings to be equal between both groups and, subsequently, by setting the factor loadings under the second-order factors. Even though the increase in the CFI was always lower than 0.01 (indicating that there was no significant deterioration of the adjustment values), the revision of the indices in the LM test suggested releasing certain restrictions. Some factor loadings were noninvariant between public and private employees. Following recommendations [65], it was possible to continue with the analysis of the invariance by starting from a partial metric invariance (information concerning the released restrictions is available by request from the corresponding author). See Table 3 for the results of the specifications performed on the measurement model.

In the first step of the scalar invariance verification (rows 7–10 of Table 3), the restriction of the intercepts of the set of indicators was tested (forcing equality between both groups), and the model was re-specified according to the suggestions derived from the LM test, and its adjustment was checked. Finally, equality restrictions were established for the latent factor intercepts in both groups. Once more, the results of the LM test led to the re-specification of the model, although CFI did not change by a value greater than 0.01, confirming partial scalar invariance and the possibility of comparing latent means of both groups [65]. To test the latent mean differences across three groups, the mean factor structure model was created. In EQS, a path was set from a constant (V999) to each of the first-order latent factors. As public organizations are considered the reference group, the averages of the first-order factors were set at 0.00 for this group of employees. For comparison of the averages in the second-order factors, paths were added from the constant (V999) to these factors, and again, the average values in these factors were set to 0.00 for employees of public organizations. Table 4 shows the significant differences in factor means and the magnitude of these differences as expressed by Cohen's *d* index. Only some of the first-order factors in the model differed. Specifically, employees in private organizations experienced higher levels of satisfaction with the environment and with legal aspects than did employees of public organizations, although the size of the effect was minuscule. Regarding engagement, employees of private organizations showed lower levels of engagement than did those of public organizations. The effect size was small (*d* = 0.29). No differences were found in the latent means of the other factors.

**Table 4:** Results of structured means analyses between private vs. public (public workers as the referent group).

Factor	Factor intercept (*SE*)	*z*-value	*d*
Engagement	−0.254 (0.054)	−4.69***	0.29
Satisfaction with environment	0.139 (0.070)	1.99*	0.13
Satisfaction with the legal aspects	0.237 (0.113)	2.09*	0.15

Note: Latent means for public organizations employees set to zero; only significant differences are included; *d* Cohen indicator of the effect size. *SE* = standard error.

*p < 0.05; *** *p* < 0.001.

### 3.3 Phase 2. Multigroup structural model analysis

Based on the theoretical aspects discussed in the introduction, a mediational model proved to have a good fit, where AL was positively related to employees’ levels of vigor at work, engagement, and satisfaction with the environment, satisfaction with superiors, and satisfaction with legal aspects. At the same time, vigor at work mediated the relationship between AL and engagement, and engagement levels were positively related to the three facets of satisfaction. Thus, engagement was also a variable that mediated the effects of both AL and vigor at work on the three facets of satisfaction.

**Fig. 1:**
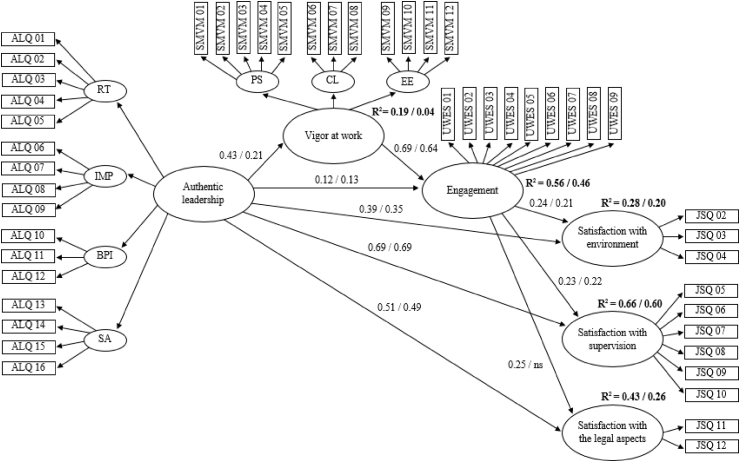
Structural model. Note: To simplify the graph, the factor loadings and the first-order factor loadings are not included. ns indicates the non-significant path. The private organizations’ coefficients values are on the left of the bar and of public organizations are on the right.

To test whether the structural paths differed between employees of private and public organizations, the model was adjusted in a multigroup analysis. The results, as well as subsequent analyses with additional restrictions built on this baseline model, are listed in Table 5. In the second step, constraints derived from the calculation of the partial metric invariance of the previous phase were introduced into the model. The results derived from the increase in S-Bχ^2^ (with nonsignificant differences) and changes in the CFI global adjustment index (<0.01) indicated an optimal fit of the model to the data with no deterioration as a result of the restrictions. Finally, restrictions related to structural paths were established to investigate possible differences between the groups in the analysis. Although the model fit was still optimal, the revision of the LM test results indicated differences in two paths of the model. Fig. 1 summarizes the model with structural paths for groups of workers in private and public organizations after the restrictions suggested by the LM test were released. The value associated with the path that related the levels of engagement to satisfaction with legal aspects differed significantly between the two groups. Specifically, this path was not significant for public organizations. Another significant difference affected the pathway that positively related AL to employees’ levels of vigor at work. The effect was greatest in the context of private organizations.

**Table 5:** Fit indices for structural model related to multigroup analyses invariance tests.

	S-Bχ^2^	*df*	RMSEA (90% CI of RMSEA)	CFI	ΔS-Bχ^2^^a^	ΔCFI
Structural model	4668.30	2128	0.048 (0.046–0.050)	0.912		
Partial metric invariance	4714.82	2166	0.048 (0.046–0.050)	0.912	44.11 (Δ*df* = 38)	0.000
Partial metric and structural path invariance	4733.73	2175	0.048 (0.046–0.050)	0.911	63.71 (Δ*df* = 47)	0.001

Note: CFI = comparative fit index; CI = confidence interval; *df* = degrees of freedom; RMSEA = root mean-square error of approximation; S-Bχ^2^ = Satorra–Bentler χ^2^ statistic.

a Corrected value.

Finally, Fig. 1 also shows the percentages of explained variance (R^2^) obtained for each of the dependent variables of the structural model for the public and private sectors. All of the R^2^ coefficients of these variables were higher in the group of workers from private organizations.

## 4 Discussion

This study aimed to analyze the mediating role of vigor at work and engagement at work between AL and different aspects of job satisfaction in the context of a *trans*-organizational model that focused on the type of organization: private or public.

Initially, in the private context, engagement levels were lower than in the public context, and thus, employees may consider exiting the organization, which is an ongoing problem in human resources management [70]. The motives for turnover and thus tactics to improve the retention of staff are closely related to leadership development. Moreover, AL has been shown to create a feeling of personal and social identification in followers and to establish an elevated moral standard and high values, which generates positive states [71]. Thus, addressing the importance of the AL style as an antecedent of satisfaction as a multidimensional construct is necessary [72]. Our study goes further by considering the mediating role that vigor at work and engagement play in several aspects of satisfaction.

Overall, the study hypotheses were confirmed. Specifically, results for hypothesis 1 showed that vigor at work partially mediated the relationship between AL and work engagement. Following the JD-R theory [28] and the suggestions of Xanthopoulou et al. [42]; a job resource, such as AL, can promote a personal and affective resource such as vigor at work and enhance the level of work engagement. This result confirms the hypothesis concerning the complementarity of both constructs [46,48], as work engagement is increased through levels of vigor at work (H1). This result allows us to propose a new perspective in explaining the link between AL and work engagement. However, sector-specific differences were found in the effects of vigor (H3). Specifically, AL had a greater positive effect on the level of vigor at work in the private sector. Thus, private organizations should consider leadership style to be an important factor due to the effect that perceived levels of AL have on employees’ attitudes and behaviors [73], which affect the level of vigor at work to a greater extent, given that this factor is a more relevant resource in the context of private organizations. Similar to other studies, we found direct implications of AL on different aspects of job satisfaction [74,75], but also indirect implications through vigor and engagement at work (H2). In contrast to other studies that have found correlations between job satisfaction and work engagement [76] or even considered work engagement as an antecedent [37,77], this study included vigor at work as another variable of interest in the relationship. However, further differences were found in the context of one aspect of job satisfaction between both sectors (H3). Specifically, work engagement was not significantly related to satisfaction with legal aspects in the context of public organizations. Although this dimension emphasizes the means and procedures for negotiating benefits and the degree of compliance [59], governmental policies concerning management and job security and stability are inherent to the public sector and differ from those of the private sector [78] and thus cannot determine work engagement in the public sector. In this case, the leader also has a great impact on increasing the engagement and satisfaction of employees. They cannot determine the wage or other extrinsic motivators but can emphasize intrinsic motivators [79]. Although the effect was not significant, employees in the public sector emphasize the importance of having a good relationship with the leader (see items in Table 2). Most likely, politicians and syndicates can have a higher influence on public organization human resources practices than on such practices in private organizations [80].

This transorganizational study contributes to our knowledge in several ways. First, it allows us to ascertain the implications of job and personal resources on engagement. Second, these resources promote changes in workers’ job satisfaction, and thus, these resources play a prevailing role within the model [81]. Third, this study helps to address the differences between public and private organizations, specifically in terms of their levels of vigor at work and satisfaction with legal aspects.

### 4.1 Limitations and strengths

The first limitation of this study stems from the cross-sectional design which suggests caution when proposing mediation effects between the study variables, as there is no temporal precedence between the measured variables [82]. The development of future longitudinal studies may contribute to overcoming this issue.

Another limitation has to do with the sampling method used. In this case, the percentage of men and women differed depending on the public or private nature of the organization. However, the higher proportion of women in public organizations is representative of the global proportion in Spain and also in other countries [83]. In a similar vein, Spanish private and public organizations may have similarities but also differ from other countries organizational contexts, and thus, the results could also differ. This may imply a certain bias in the generalization of the results. Further, in spite of the good fit obtained by the multigroup structural model, alternative models were not analyzed, which does not rule out the possibility that other models also fit properly. Finally, the use of self-reported measures does not allow us to discard the presence of social desirability bias in the participants’ responses.

However, the study also has strengths. First, no previous studies have analyzed the relationship between vigor at work and work engagement as mediators of the relationship between AL and satisfaction outcomes within the public and private sectors; thus, further studies should confirm the proposed model. Second, most studies, including those concerning engagement or vigor at work, have analyzed the relations of those factors to AL in the private sector. Despite recent attention from public organizations on ways of improving the performance of public servants, scientific public management research, although growing, remains limited [84]. Additionally, the absence of studies analyzing how public organizations change when incorporating practices taken from private organizations [85] could explain why our results indicated fewer differences than expected. Thus, it is important to deepen this comparison and to differentiate the results according to the sector. For future studies, hybrid organizations (public/private) could be considered to analyze whether these practices explain the different results [86].

## 5 Conclusion

This cross-sectional and cross-sectoral study provides interesting insights and results. From JD-R theory [28], the results showed that vigor at work and work engagement were important variables to explain the AL-job satisfaction relationship in both private and public organizations. Nevertheless, the relationship between work engagement and the dimensions of job satisfaction was different in both types of organizations. Specifically, work engagement was significantly related to satisfaction with the environment and supervision in both organizational contexts, but not with legal aspects in public organizations. Previously, no studies had analyzed the relationship between vigor at work and work engagement as mediators of the relationship between AL and satisfaction outcomes within the public and private sectors; and most studies including either engagement or vigor had analyzed their relations with AL in the private sector. Despite the recent attention from public organizations to improve the performance of public servants, scientific public management research is growing but limited [87]. Thus, this paper could be of great interest to scientists, practitioners and governments, because the findings of this study can facilitate decision-making concerning the development of intervention programs in light of specific variables (e.g., leadership style), including increasing levels of vigor, engagement, and dimensions of satisfaction that should be improved. Finally, interventions should differ depending on their organizational context and should focus on engagement in private organizations and on satisfaction in public organizations.

## Author contribution statement

Daniel Cortés-Denia: Performed the experiments; Contributed reagents, materials, analysis tools or data; Wrote the paper.

Octavio Luque-Reca: Performed the experiments; Analyzed and interpreted the data; Contributed reagents, materials, analysis tools or data; Wrote the paper.

Esther Lopez-Zafra: Conceived and designed the experiments; Performed the experiments; Contributed reagents, materials, analysis tools or data; Wrote the paper.

Manuel Pulido-Martos: Conceived and designed the experiments; Performed the experiments; Analyzed and interpreted the data; Contributed reagents, materials, analysis tools or data; Wrote the paper.

## Funding statement

This study was supported by the Spanish Ministry of Science and Innovation through the project PID2020-116521RB-I00, funded by CIN/AEI/10.13039/501100011033, and by a predoctoral fellowship (FPU18/00302).

## Data availability statement

The data that support the findings of this study are available from the corresponding author upon reasonable request.

## Declaration of interest statement

The authors declare that they have no known competing financial interests or personal relationships that could have appeared to influence the work reported in this paper.
